# Cholangiocarcinoma Presenting as Humoral Hypercalcemia of Malignancy: A Case Report and Literature Review

**DOI:** 10.7759/cureus.6481

**Published:** 2019-12-27

**Authors:** Burak Erdinc, Preethi Ramachandran, Ruchi Yadav, Sonu Sahni, Gardith Joseph

**Affiliations:** 1 Internal Medicine, Brookdale University Hospital and Medical Center, Brooklyn, USA; 2 Oncology, Brookdale University Hospital and Medical Center, Brooklyn, USA; 3 Hematology and Oncology, Brookdale University Hospital and Medical Center, Brooklyn, USA; 4 Oncology, Mont Sinai Medical Center, Brooklyn, USA

**Keywords:** cholangiocarcinoma, humoral hypercalcemia of malignancy, parathyroid hormone related peptide, parathyroid hormone, paraneoplastic syndrome, cholangiocarcinoma

## Abstract

Humoral hypercalcemia of malignancy (HHM) is most commonly encountered in squamous cell carcinoma (SCC) of different organs, and It is characterized by elevated parathyroid hormone-related peptide (PTHrP) levels. It may be seen as a manifestation of cholangiocarcinoma (CCC) at presentation and later in the course of the disease. However, HHM due to intrahepatic cholangiocarcinoma is a rare association and is associated with a poor prognosis.

We herein report a case of hypercalcemia presenting as the first manifestation of an underlying rare variant of intrahepatic cholangiocarcinoma. Our patient is a 57-year-old male who presented to the emergency room with severe symptoms of constipation and polyuria and was admitted to the hospital for symptomatic hypercalcemia. He was found to have a hypermetabolic 15 cm liver mass, abdominal lymph nodes on imaging, which was subsequently diagnosed as adenosquamous cholangiocarcinoma by liver biopsy. This necessitated an urgent inpatient treatment with gemcitabine/cisplatin combination chemotherapy to control the aggressive nature of the malignancy. However, he deteriorated and expired after three months of his diagnosis.

Adenosquamous cholangiocarcinoma is a very rare variant of a liver tumor. It develops due to squamous metaplasia of an underlying cholangiocarcinoma and usually has aggressive clinicopathological features. HMM is a life-threatening, yet unrecognized, phenomenon of cholangiocarcinoma, which represents a poor prognostic marker. Prompt recognition of this complication is important for preventing serious complications associated with hypercalcemia and to improve the quality of life of these patients.

## Introduction

Hypercalcemia of malignancy is seen in up to 30% of all cancers via four different mechanisms: humoral hypercalcemia of malignancy (HHM), local osteolytic hypercalcemia, excess 1,25(OH)2D secretion, and ectopic parathyroid hormone secretion [1]. Parathyroid hormone-related peptide (PTHrP) secretion is the most common mechanism (80%) causing hypercalcemia of malignancy [1-2]. Even though HHM theoretically can be seen in any type of cancer, it is very rarely seen with cholangiocarcinoma, as in our case [3]⁠.

## Case presentation

A 67-year-old Hispanic male presented to the emergency department with gradually worsening complaints of generalized body weakness, difficulty sleeping, abdominal pain, increased thirst with urinary frequency, and constipation for the last two months. He had a past medical history of a fatty liver secondary to alcohol use and a four-pack per year smoking history. The initial physical examination was unremarkable with normal vital signs except for hepatomegaly. The laboratory data on admission are summarized in Table 1.

**Table 1:** Initial laboratory investigations

	Normal Range	Result
White Blood Cell Count	4.10 - 10.10 10x9/L	17.60 (H)
Hemoglobin	12.9 - 16.7 g/dL	12.7 (L)
Mean Corpuscular Volume	80.8 - 94.1 fL	86.5
Platelet Count	153 - 328 10x9/L	422 (H)
Absolute Neutrophil Count	1.40 - 6.80 10x9/L	12.80 (H)
Absolute Lymphocyte Count	1.10 - 2.90 10x9/L	2.60
Absolute Monocyte Count	0.20 - 1.00 10x9/L	1.20 (H)
Absolute Eosinophil Count	0.00 - 0.40 10x9/L	0.80 (H)
Absolute Basophil Count	0.00 - 0.10 10x9/L	0.20 (H)
International Normalized Ratio	0.70 - 1.20	1.08
Blood Urea Nitrogen	9.0 - 20.0 mg/dL	18.0
Creatinine	0.66 - 1.25 mg/dL	0.94
Sodium	133 - 145 mEq/L	134
Potassium	3.5 - 5.1 mEq/L	4.1
Chloride	98 - 107 mEq/L	96 (L)
CO_2_	22 - 30 mEq/L	31 (H)
Calcium	8.4 - 10.5 mg/dL	15.2 (H)
Phosphorus	2.5 - 4.5 mg/dL	2.9
Total Protein	6.3 - 8.2 g/dL	6.4
Albumin	3.5 - 5.0 g/dL	3.4 (L)
Total Bilirubin	0.2 - 1.3 mg/dL	0.5
Alanine Aminotransferase	21 - 72 U/L	29
Aspartate Aminotransferase	17 - 59 U/L	20
Alkaline Phosphatase	38.0 - 126.0 U/L	141.0 (H)
Gamma-Glutamyl Transferase	15 - 73 U/L	276
Magnesium	1.6 - 2.3 mg/dL	1.4 (L)
Parathyroid Hormone0Related Protein	14-27 pg/ml	82(H)
Alfa Fetoprotein	<6.1 ng/mL	2.2
Cancer Antigen 19-9	<34 U/mL	140 (H)
Carcinoembryonic Antigen	0.0 - 2.4 ng/mL	1.5
Intact Parathyroid Hormone	14 - 64 pg/mL	2 (L)
Vitamin D,1,25-(OH)2	18 - 72 pg/mL	31

**Figure 1:**
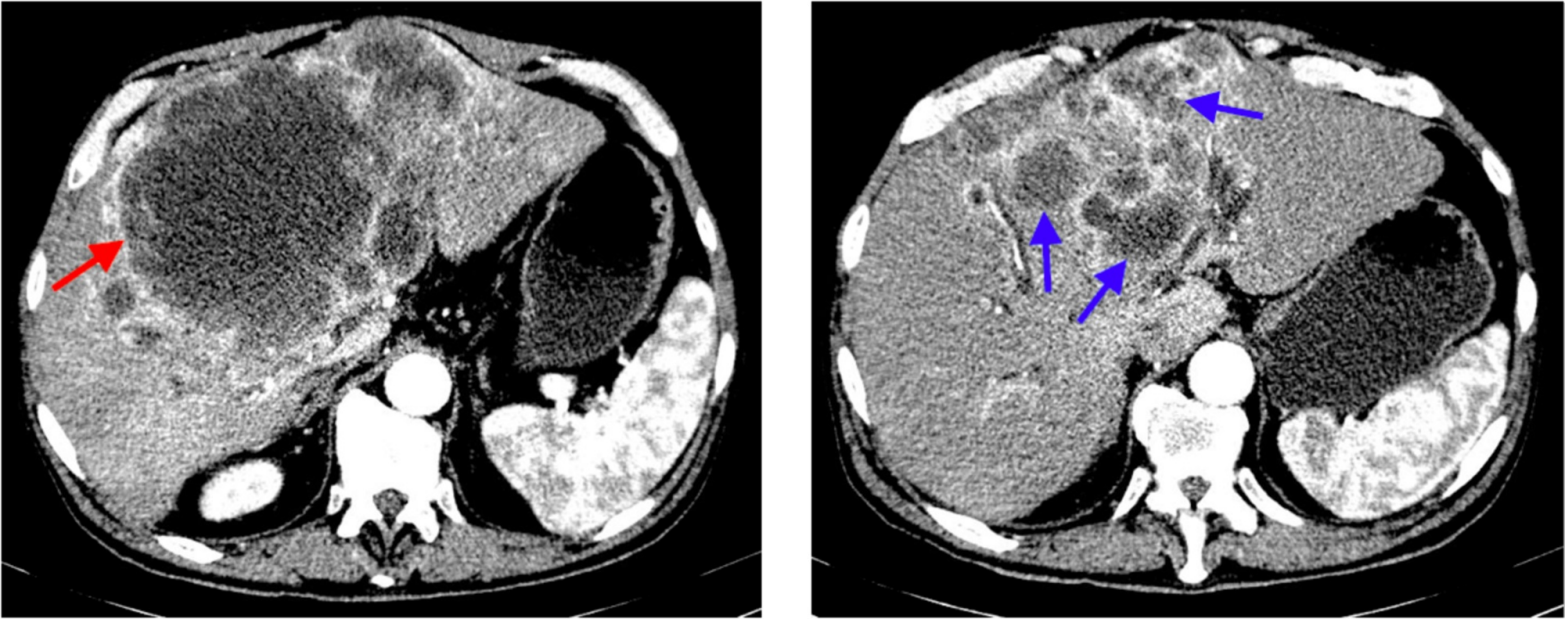
Computerized tomography of the abdomen with intravenous contrast showing a large, complex-appearing mass within the liver appearing as multifocal lesions with a large central hypodense lesion (red arrow) and numerous peripheral satellite lesions (blue arrows); the largest component of the mass measured 19.1 x 14.7 cm

The initial laboratory evaluation showed leukocytosis (white blood cell count of 16.60 10x9/L), normal kidney function, elevated corrected calcium level of 15.5 mg/dL, elevated alkaline phosphatase level of 141 U/L (normal range 38.0-126.0 U/L), and normal phosphorus level of 2.9 mg/dl (normal range 2.5 - 4.5 mg/dl). Intact parathyroid level was found to be low at 2 pg/ml (normal range 14-64 pg/ml). However, parathyroid hormone-related peptide level (PTHrP) was high at 82 pg/ml (normal range 14-27 pg/ml). His other laboratory values included low vitamin D,25-Hydroxy level of 13.4 ng/ml (normal range 30-100 ng/ml), carcinoembryonic antigen (CEA) level of 1.5 ng/mL (normal range: <5.0 ng/mL), carbohydrate antigen (CA) 19-9 level of 140 U/mL (normal range: <34 U/mL), α-fetoprotein (AFP) level of 1.6 ng/mL (normal range: < 6.1 ng/mL), and serum protein electrophoresis was within normal limits. He had negative viral serology for hepatitis B, hepatitis C, Epstein Barr Virus, cytomegalovirus, and human immunodeficiency virus. A computerized tomography (CT) scan of the abdomen and pelvis with intravenous contrast was performed to evaluate the etiology of abdominal pain, which revealed a large complex appearing mass within the liver appearing as multifocal lesions with a large central hypodense lesion and numerous peripheral satellite lesions; the largest component of the mass measured 19.1 x 14.7 cm (Figure 1). There was also mild intrahepatic bile duct dilatation, most likely due to mass-effect on the porta hepatis, and haziness within the fat inferior to the liver, which was concerning for peritoneal carcinomatosis.

**Figure 2:**
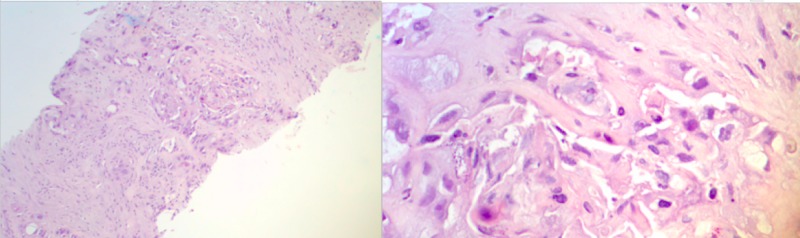
Liver core biopsy, H & E stain, poorly differentiated adenocarcinoma with squamous features (10X on the left and 50X on the right) H & E: hematoxylin and eosin

**Figure 3:**
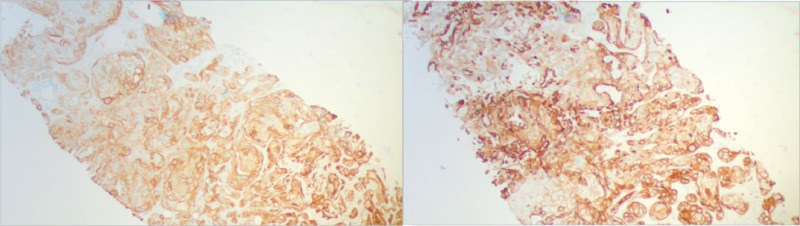
Liver core biopsy, 10X. Immunohistochemical stain CK19 positive, favor pancreatic-biliary primary (left). Liver core biopsy, 10X. Immunohistochemical stain CK5/6 positive, favor squamous features (right)

The patient was admitted to the hospital and received treatment with intravenous fluid resuscitation, calcitonin, and zoledronic acid for hypercalcemia of malignancy. Further evaluation with positron emission tomography-computed tomography (PET-CT) reported extensive hyper-metabolic activity in the liver, which corresponded to a large, complex cystic mass and multiple satellite lesions on CT images, consistent with malignancy. In addition, It revealed hypermetabolic, small, and mildly enlarged upper abdominal lymph nodes, mildly hypermetabolic activity in the omentum/peritoneum, and ascites; all these findings were suspicious for metastatic disease. Lastly, there were small scattered pulmonary nodules, which were below the size resolution of PET imaging. CT-guided biopsy of the liver was performed, and pathology showed poorly differentiated adenocarcinoma with squamous features supportive of pancreaticobiliary as primary (Figure 2). Tumor cells were positive for CEA, CK19, P63, CK5/6, focally positive for CK7, and negative for CK20 and hepatocyte stains (Figure 3).

Chemotherapy with cisplatin and gemcitabine was started after the diagnosis was confirmed. However, the patient had recurrent admissions with worsening symptoms and uncontrolled hypercalcemia. His course was also complicated further with acute kidney injury, cholestatic hyperbilirubinemia, and pancreatitis and required placement of percutaneous transhepatic cholecystostomy (PTC). The patient’s condition further deteriorated, and he expired within three months of his diagnosis.

## Discussion

Cholangiocarcinoma is a very aggressive tumor and comprises about 3% of all gastrointestinal malignancies [4]⁠. Studies on this rare type of tumor are only limited to case reports or small case series [2,5-6]⁠. Primary sclerosing cholangitis (PSC), fibropolycystic liver disease, hepatolithiasis, chronic viral hepatitis, and a few genetic conditions, such as Lynch syndrome, BRCA-associated protein-1 (BAP1) tumor predisposition syndrome, cystic fibrosis, and biliary papillomatosis are among the common risk factors for cholangiocarcinoma [7]⁠. Toxic exposure to thorotrast (previously used radiocontrast agent) [8]⁠, parasitic infections with Clonorchis and Opisthorchis [9]⁠, and human immunodeficiency virus (HIV) infection [10]⁠ have also proved to have a relationship with cholangiocarcinoma.

Adenosquamous cholangiocarcinoma (ASC) is a rare variant of cholangiocarcinoma, which was first described by Barr and Hancock in 1975 [11]⁠ and consists of malignant glandular and squamous components. Tumor cells have both mucin-producing glandular structures as in adenocarcinoma and irregularly shaped solid nests of polygonal cells with distinct cellular borders, eosinophilic cytoplasm, varying degrees of keratinization, and intercellular bridges as in squamous cell cancer [12-13]⁠. Cholangiocarcinoma with squamous features have been previously described as ASC but also as mucoepidermoid carcinoma (mucus-producing cells and squamoid cells without keratin formation) and adenoacanthoma (squamous metaplasia) [6,14]⁠. The prognosis of ASC is extremely poor even with surgery and most patients are not candidates for surgery and chemotherapy at presentation [2,6,12]⁠. Nakajima and Kondo [2]⁠ evaluated 11 patients with ASC and compared their prognosis with 82 cholangiocarcinoma patients with adenocarcinoma features and found that the mean overall survival was four months in ASC patients versus 6.9 months in the latter.

HHM is caused by PTH-related peptide (PTHrP) by malignant tumors, such as squamous cell carcinoma, renal cell carcinoma, ovarian cancer, endometrial cancer, lymphoma, and breast cancer [1]⁠. PTHrP has different identified functions, such as proliferation and differentiation in chondrocytes [15], ⁠regulation of calcium level in the placenta [16]⁠, calcium reabsorption in renal cells, resorption of calcium from osteocytes [17],⁠ and browning of adipose cells and cachexia [18-19].⁠ PTHrP secreting cancer cells can be detected, for diagnostic and research purposes, with immunohistochemical stains (PTHrP antibody stain). Even though HMM has been previously described in different types of cancers, it is an unrecognized phenomenon of cholangiocarcinoma and associated with a very poor prognosis [20]⁠. For this reason, the early detection and treatment of HHM is essential. The treatment of HHM should include immediate treatment with intravenous hydration and calcitonin and long-term treatment with bisphosphonates and denosumab. Hemodialysis is usually reserved for patients with severe hypercalcemia.

## Conclusions

Our case is an example of metastatic adenosquamous cholangiocarcinoma without bony metastasis, presenting as hypercalcemia, secondary to PTHrP levels confirming the diagnosis of HHM. Adenosquamous cholangiocarcinoma, being a very rare variant of cholangiocarcinoma presents with HHM, which is also a very rare presentation, as it is usually associated with other types of cancers. Physicians should be aware of this rare presentation of cholangiocarcinoma, as prompt recognition of this atypical presentation can prevent the serious complications associated with hypercalcemia and improves the quality of life of these patients.
